# A Study to Assess Catastrophic Household Expenditure on Childhood Illness in an Urban Slum in Bijapur

**DOI:** 10.4103/0970-0218.58394

**Published:** 2009-10

**Authors:** Shailaja Sharabasappa Patil, Aditya Suryabhan Berad, Mahabaleshwar Mahantappa Angadi

**Affiliations:** Department of Community Medicine, Shri B M Patil Medical College, Bijapur, Karnataka, India

**Keywords:** Catastrophic, childhood illness, expenditure

## Abstract

**Objective:**

In this study, the various factors determining the out-of-pocket expenditure on child health care by households are discussed to answer the following questions: How much are households currently spending on child health care? Is there any role of socio-economic status of households on expenditure on child health care? What percentage of their income is spent on child health care and is it catastrophic?

**Materials and Methods:**

Four slums with a total a population of 7000 were selected for this study. Households where there is history of illness/ sickness in children under 5 years in last one month were included in the study.

**Results:**

There were a total of 218 episodes of child illness in the households. The household's belonging to socio- economic class I and II had higher spending on child's illness per episode as compared to households of socio- economic class III, IV, and V. Socioeconomic status was the key determinant of health care expenditure.

**Conclusion:**

In this study, it has been found that almost all the households suffered from catastrophic health expenditure.

## Introduction

The relationship between poverty and ill-health is indisputable. There is more recent evidence to show the effect of illness on poverty.(1) Even relatively small expenditure on health can be financially disastrous for poor households. This is because almost all their financial resources are used for basic needs and they are thus less able to cope with even very low expenditure compared to richer households.(2) In developing countries, high out of pocket payment, an absence of risk pooling mechanism in health financing systems, and high level of poverty can result in catastrophic health expenditure.(3)

India has adopted the tax-based model of financing health care. However, decades of under-funding have resulted in poor infrastructure, vacant posts, and poor quality health care. This makes the patients seek private health care for their needs, with its associated out-of-pocket payments. Studies show that about a quarter of Indians who are hospitalized are impoverished because of the medical costs.(4)

We tried to study the following factors determining the out-of-pocket spending on child health care by households to answer following questions: How much are households currently spending on child health care? Is there any role of socio-economic status of households on expenditure on child health care? What percentage of their income is spent on child health care and is it catastrophic?

## Materials and Methods

Four slums with total population of 7000 adjoining urban health training centre were selected for the study. A total of 658 children under the age of five years reside in the area. All the households having children aged less than 5 years were visited. Only those households where there is history of illness/sickness in children under 5 years in last one month were included in the study to minimize the recall bias. The head of households and the mothers of the sick children were interviewed by using pretested and prestructured questionnaire. Questions were asked on the type of illness the child was suffering, place where treatment was sought, and information on expenditure incurred. The socio- economic classification of household's was done using modified Prasad's scale according to current consumer price index. The data so collected was analyzed using EPI6 software in the department of Community Medicine.

## Results

Data from 190 households were collected and analyzed, where at least one child aged less than 5 years was ill in the past one month period. There were total 218 episodes of child illness in the households. The mean duration of each episode was 4.6 days. 158 episodes were treated at private hospitals, 53 episodes at public hospitals whereas for 7 episodes no treatment was sought. Out of 190 households, only 13 households were having some form of health insurance. Thirty households (16%) had some plan for health insurance.

Table 1 provides the results of spending on child illness. The household's belonging to socio- economic class I and II had higher spending on child's illness per episode as compared to households of socio- economic class III, IV, and V. This difference in the level of spending according to different socio- economic class of household's was found to be significant in all sub groups of expenditure. This difference may be due to the fact that socio- economic class I and II availed services from private practitioners where the hospital fees and drug prescription amounts were considerably higher.

**Table 1:** Out of pocket spending on child's illness per episode according to socio-economic class of households

Socio-economic class	Hospital fees in INR median (95% CI)	Spending on drugs in INR median (95% CI)	Spending on travel in INR median (95% CI)	Total median spending in INR (95% CI)
I	75 (20–150)	150 (65–450)	20(10–40)	327 (145–1730)
II	40 (15–100)	100 (50–200)	20 (0–40)	140 (110–400)
III	25 (15–50)	70 (50–100)	20 (0–30)	120 (80–160)
IV	20 (5–40)	52 (30–82)	20 (0–20)	100 (71–145)
V	20 (0–40)	55 (20–90)	20 (10–35)	105 (72–150)
*P* value	*P*<0.01	*P*<0.001	*P*<0.01	*P*<0.01

## Discussion

Our results showed that socio-economic status was the key determinant of health care expenditure. These findings are similar to that reported in multicountry analysis of catastrophic household healthcare expenditure.(3) The poorest, that is, socio-economic class V households spent a total of Rs 105 (95%CI 72-150) per episode of child illness. The NSS fifty-second round(5) data shows that poorest spent Rs 77 per episode in rural areas and Rs 95 per episode in urban areas. There are some hidden costs even if the patients attend the public sector hospitals. These are mainly on travel expenses and wages lost if the child is admitted and on drugs that are bought from market. Health expenditure has been defined as catastrophic if 5–20% of total household income is spent on health care.(5–9) In our study, it has been found that almost all the households suffered from catastrophic health expenditure [Table 2]. Protection of interest of the poorest class households who suffered catastrophic expenditure should be addressed in policy formulations to ensure better access to health services and higher degree of financial protection against the economic impacts of illness on the family.

**Table 2:** Percentage of total out of pocket health care spending on child's illness out of total income of households

Socio-economic class of households	Number of households studied	Total income of households (A) (mean ± SD)	Total household expenditure on child health care (B) (mean ± SD)	Percentage of B out of A (%)
I	12	187500 (15625 ± 10394)	33680 (2086 ± 5333)	17.9
II	31	203050 (6550 ± 3200)	19065 (615 ± 1509)	9.3
III	55	237000 (4309 ± 1749)	13275 (241 ± 462)	5.6
IV	76	197850 (2603 ± 1154)	9817 (129 ± 142)	4.96
V	16	19725 (1232 ± 426)	1975 (125 ± 81)	10.0

## Conclusion

We conclude that poorest members of community incurred catastrophic health expenses. Our findings have important policy implications and can be used to ensure better access to health services and a higher degree of financial protection for low-income groups against economic impact of illness.
